# Effects of growth hormone administration on luteinizing hormone secretion in healthy older men and women

**DOI:** 10.14814/phy2.13516

**Published:** 2017-12-06

**Authors:** Ranganath Muniyappa, Shannon D. Sullivan, Sri Harsha Tella, Brent S. Abel, S. Mitchell Harman, Marc R. Blackman

**Affiliations:** ^1^ Diabetes, Endocrinology, and Obesity Branch National Institute of Diabetes and Digestive and Kidney Diseases NIH Bethesda Maryland; ^2^ Department of Endocrinology Georgetown University and Washington Hospital Center Washington District of Columbia; ^3^ Endocrinology Section Department of Medicine Phoenix VA Health Care System Phoenix Arizona; ^4^ Division of Endocrinology and Metabolism Department of Medicine Johns Hopkins University School of Medicine Baltimore Maryland; ^5^ Research Service Veterans Affairs Medical Center Washington District of Columbia

**Keywords:** Aging, Growth hormone, Luteinizing hormone, Pulsatility

## Abstract

The known interactions between the somatotropic and hypothalamic‐pituitary‐gonadal (HPG) axes have not been well delineated in older individuals. Aging‐associated decline in insulin like growth factor‐1 (IGF‐1) levels has been proposed to play a role in reproductive senescence in animals. However, the effects of GH on LH secretion are unknown in older individuals. Our objective was to determine whether GH modulates LH secretion or levels of sex steroids (SS) in healthy older (ages 65–88 years) men (*n* = 24) and women (*n* = 24) with low‐normal plasma IGF‐1 levels. In a double‐masked, placebo‐controlled (*n* = 24), randomized study, we evaluated the effects of GH (*n* = 24, 20 *μ*g/kg sc 3×/week) for 26 weeks on nocturnal LH secretory dynamics [(8 pm to 8 am, Q20) min sampling and analyzed by multiparameter deconvolution algorithm]. Indices of LH secretion [frequency, mass per burst, pulsatile production rate, and approximate entropy (ApEn)] and fasting serum IGF‐1, SHBG, and SS (TT, fT, or E2) were measured. At baseline, all indices of LH secretion (frequency, mass per burst, pulsatile production rate) were inversely (*P* < 0.05) related to IGF‐1, but not to mean nocturnal serum GH concentrations. GH administration for 26 weeks increased serum IGF‐1, but exerted no significant effects on LH secretory dynamics, or concentrations of SSs (TT, fT, or E2) or SHBG in older women or men. These data suggest that GH‐mediated increases in IGF‐1 do not modulate the HPG axis in older individuals.

## Introduction

The hypothalamus, pituitary gland, and gonads with feedback loops constitute the hypothalamic‐pituitary‐gonadal (HPG) axis (Veldhuis 2008). Gonadotropin‐releasing hormone (GnRH) released in a pulsatile manner into the hypothalamo‐pituitary portal system stimulates the secretion of luteinizing hormone (LH) and follicle stimulating hormone (FSH) from the pituitary gonadotrophs. Circulating LH and FSH act on the gonads to release sex steroid (SS) hormones which then negatively feedback onto the pituitary and hypothalamus to inhibit GnRH and LH secretion. In females, SSs such as estradiol (E2) and progesterone (P) or peptide hormones such as inhibin negatively regulate the secretion of GnRH and LH (by SSs), and FSH (by inhibin and E2) (Hall 2015). However, GnRH pulse frequency varies depending on the phase of the ovarian cycle. The follicular phase is characterized by high‐frequency LH pulses due to the positive feedback from increasing E2 concentrations released by the preovulatory follicles (Hall 2015) and the luteal phase is characterized by the low‐frequency LH pulses.

The somatotropic axis consists of growth hormone (GH), insulin‐like growth factors (IGF‐1 and ‐II), IGF binding proteins, and their respective receptors. Interactions between the somatotropic and HPG axes are well‐known. Hypothalamic GnRH neurons as well as pituitary gonadotrophs express IGF‐1 receptors, providing a potential mechanism for the regulation of gonadotrophin secretion by the GH/IGF‐1 axis (Wilson 2001; Gutierrez et al. 2007; Wolfe et al. 2014). Central actions of IGF‐1 play an important role in the regulation of reproductive function in rodents (Hiney et al. 1996; Divall et al. 2010). Restoration of hypothalamic IGF‐1 by gene therapy prevents the age‐related decline in central IGF‐1 and reproductive function in rodents (Rodriguez et al. 2013). In humans, aging is associated with decreased production of IGF‐1 (Corpas et al. 1993) and SS (Lamberts et al. 1997), higher sex hormone binding globulin (SHBG) levels (Gray et al. 1991; Burger 1999; Muller et al. 2003), and altered GnRH/LH pulsatility that are independent of changes in SS levels (Hall et al. 2000; Veldhuis 2008). Whether declining IGF‐1 levels play a role in reproductive senescence in humans remains unknown.

In young individuals, GH contributes to the regulation of puberty and fertility via HPG axis stimulation through changes in the levels of SS and/or gonadotropin secretion (Wilson 2001; Chandrashekar et al. 2004; Giampietro et al. 2009). Specifically, GH acts on its receptors in the ovary to promote steroidogenesis and gametogenesis via gonadotropin‐independent stimulation of P and E2, inhibition of follicular apoptosis, and upregulation of ovarian LH receptors (Bartke 1999; Hull and Harvey 2001; Bachelot et al. 2002). Treatment with GH has been shown to reinstate normal ovarian activity in GH insufficient girls and women, who suffer from delayed puberty, abnormal menstrual cycling, and infertility (Spiliotis 2003). IGF‐1 receptors in Leydig and Sertoli cells of the testes and in primary spermatocytes play an important role in normal testicular steroidogenesis (Chandrashekar et al. 2004). Healthy men treated with exogenous GH for 3 weeks demonstrate heightened Sertoli cell function (Andreassen et al. 2013). Not surprisingly, based on the stimulatory effect of GH on SS production in young individuals, GH deficiency and GH resistance results in delayed puberty in boys and girls (Laron 1999), suggesting a permissive relationship between the somatotropic and reproductive axes**.** GH administration to women with amenorrhea increases plasma E2 levels, LH pulse frequency, and reduces LH pulse amplitude (Genazzani et al. 1993). However, based on this study and in nonhuman primates (Wilson 1995) the change in LH pulsatility was attributed to ovarian feedback activity due to increases in E2 rather than a direct effect on GH/IGF‐1 on gonadotropes. In the current investigation, we examined the effects of exogenous GH administration and its associated increase in serum IGF‐1 levels on pulsatile secretion of LH in healthy older men and women with low‐normal circulating levels of IGF‐1 and SS.

## Subjects and Methods

### Study Population

The study population and protocol design have been published in detail previously (Blackman et al. 2002). At study entry, all participants were 65 years of age or older, nondiabetic based on fasting glucose levels, and healthy based on history, physical examination, routine serum chemistries, and treadmill exercise stress electrocardiogram testing. Participants did not smoke, consumed ≤ 30 grams of alcohol daily, and took no medications that interfere with GH‐IGF‐1 axis function or gonadal steroid concentrations. Of note, women had not taken any form of hormone replacement therapy for at least 3 months prior to study entry, and men had never taken testosterone (T) replacement prior to study entry. To be eligible for the study, all subjects had to have age‐related declines in circulating IGF‐1 levels, at least 1 SD below the mean for levels in healthy young (age 20–35) adults (≤230 *μ*g/L) (O'Connor et al. 1998), and men had to have serum total T levels of ≤ 16.3 mmol/L (Harman et al. 2001). The study was approved by the combined Institutional Review Boards of Johns Hopkins Bayview Medical Center and the National Institute on Aging Intramural Research Program. Each participant signed a written informed consent.

## Study protocol, hormone administration, and assays

### Study design

A detailed description of the study protocol, hormone doses used, and laboratory assays has been published previously (Blackman et al. 2002). The protocol was approved by the combined institutional review board of the Johns Hopkins Bayview Medical Center (JHBMC) and the Intramural Research Program, National Institute on Aging. Written informed consent was obtained from each participant. Briefly, a 2 × 2 factorial, placebo‐controlled, double‐dummy design was used to randomize participants to receive either GH (20 *μg*/kg sc 3x/week) plus placebo, sex steroid (transdermal estradiol plus oral medroxyprogesterone acetate in women, intramuscular injections of testosterone enanthate in men) plus placebo, GH plus SS, or placebo only. Participants were healthy women (*n* = 57) and men (*n* = 74), aged 65‐88 years. The drop‐out rate in the original study was, 4.7%. In the current substudy, we evaluated indices of LH secretion and levels of IGF‐1, sex steroids [total and free T and estradiol (E2) in women], and SHBG before and after treatment in individuals who received either GH plus placebo (GH; *n* = 12W, *n* = 12M) or placebo alone (placebo; *n* = 12W, 12M) for 26 weeks. Two women and five men in the placebo group, and one woman and five men in the GH group from the original completed cohort did not have nocturnal LH secretion evaluated and thus are not included in this analysis. Participants randomized to receive exogenous SS were excluded from these analyses due to known effects of SS on the HPG axis.

One of the secondary aims of the original study was to examine the effects of GH administration on LH secretory dynamics and SS levels. Early morning (8 am) concentrations of LH, IGF‐1, total T (TT) and free T (fT), E2 (women only), and SHBG were measured at baseline and after 26 weeks of GH or placebo administration. Nocturnal LH secretory dynamics (frequency, burst mass, pulsatile production rate, and integrated LH secretion) were also determined at baseline and after the treatment period using frequent overnight blood sampling as follows. Participants were admitted to the General Clinical Research Center of the Johns Hopkins Bayview Medical Center during the evening of day 1. At 0800 on day 2, after an overnight fast, blood samples were obtained for baseline determinations of LH, IGF‐1, TT, fT, E2, and SHBG. At 1900 h, an intravenous catheter was inserted into a forearm vein, and from 2000 h to 0800 h, blood samples (2 mL) were collected every 20 min for measurement of LH. All sera were saved at −80^°^C until assayed. Participants were discharged on the afternoon of day 3. At week 26, all baseline procedures were repeated.

### Hormone administration

Placebo or recombinant human growth hormone (rhGH) (Nutropin, Genetech Inc., San Francisco, CA) was administered as 20 *μ*g (0.055U) per kg body weight, self‐injected sc, three times per week, in the evening. Alterations in dosage due to adverse reactions have been detailed previously (Blackman et al. 2002).

### Assays

Detailed descriptions of the assays used have been published previously (Blackman et al. 2002). All samples were assayed in duplicate. Total and free testosterone concentrations were measured using an iodinated radioimmunoassay (RIA) (ICN Pharmaceuticals, Irvine, CA), with a sensitivity of 0.008 nmol/L (0.22 ng/dL). Total and free T were measured in the same assay to avoid interassay variation. Sensitivity and precision in the measurement of fT were optimized by equilibrium dialysis method, with a sensitivity of 0.4 pg/mL. Serum E2 was measured by RIA (normal range 49–199 pg/mL), with a sensitivity of 20 pg/mL. SHBG was measured by coated tube immunoradiometric assay (IRMA) (Diagnostic Systems Laboratories, Webster, TX) with a sensitivity of 5 nmol/L. Serum LH was measured by IRMA using commercial kits (Nichols Institute Diagnostics, San Juan Capistrano, CA). Sensitivity and intra and interassay CV of the LH assay were 1 mIU/mL and 3.2% and 4.5%, respectively. Serum IGF‐1 levels were measured by RIA (normal range 250–750 ng/mL), with a sensitivity of 30 ng/mL.

### Analysis of pulsatile LH secretion

A preliminary fit for the LH secretory profile was determined by a waveform‐independent deconvolution methodology (PULSE2). This was followed by a multiparameter deconvolution methodology (DECONV). The secretory parameters characterized for LH were basal secretion, secretory burst frequency (number of secretory peaks over the 12 h sampling period), mean interpulse interval, mass/burst (average amount of hormone secreted per episode), mean burst amplitude (average of calculated maximal rates of secretion for all secretory episodes), pulsatile production rate (production rate of LH during pulse per minute), total production rate, fractional pulsatile secretion, and mean and integrated 12 h concentrations.

### Approximate entropy

To evaluate orderliness of LH release, approximate entropy (ApEn) for LH was also assessed, details of which have been published previously (Gusenoff et al. 2001). Briefly, ApEn refers to the regularity or orderliness of hormone release, with a higher ApEn reflecting a more random or disordered pattern of secretion.

### Statistical analyses

Frequency distributions of all outcome variables were analyzed and log transformed where appropriate. All LH deconvolution outcomes and LH single AM samplings (LH AM) were log transformed. Baseline group differences and intervention effects on SHBG and all LH and gonadal steroid outcomes were evaluated using a sex‐specific ANCOVA, adjusted for age and group. All data are expressed as mean (95% confidence intervals, CI). Means and CIs were calculated from the log‐transformed data, as appropriate, and were subsequently back transformed (by taking the antilog of the values), resulting in geometric means and corresponding 95% CIs. Differences between treatment groups were assessed by ANCOVA performed using the General Linear Models (GLM) Procedure. The dependent variables in the ANCOVA were the changes (post − pre) in values of the outcome variable being studied. Independent variables included the subject's age, baseline value of the outcome variable, and two variables indicating treatment group (GH) and placebo. A *P*‐value of <0.05 (two‐tailed) was considered significant.

## Results

### Subject characteristics

Table 1 summarizes mean values for age and basal AM serum levels of LH, total T and fT, SHBG, E2 (women only), and IGF‐1. Age did not differ significantly by sex or treatment group, and within each sex, no significant differences in hormone levels were observed between treatment groups at baseline. At baseline, indices of LH secretion (frequency, mass per burst, pulsatile production rate) were inversely (*P* < 0.05) related to IGF‐1, but not to mean nocturnal serum GH concentrations.

**Table 1:** Hormonal characteristics at baseline and after 26 weeks of GH administration in healthy older men and women

	Men			Women		
	Placebo (*n* = 12)	GH (*n* = 12)	*P* value	Placebo (*n* = 12)	GH (*n* = 12)	*P* value
Age (baseline)	68.9 (66.8–70.9)	70.9 (67.6–74.1)		72.3 (69.3–75.1)	70.3 (67.8–72.8)	
LH, IU/L (8 am)						
Baseline	3.2 (2.6–3.9)	3.5 (2.5–4.9)		19.5 (15.0–25.3)	18.9 (15.3–23.3)	
26 Weeks	3.1 (2.4–3.9)	3.7 (2.9–4.6)		19.5 (14.7–26.0)	18.9 (15.3–23.6)	
^ ^ §Between group differences in ∆	1.13 (0.93–1.37)		0.20	1.03 (0.91–1.22)		0.56
Total Testosterone, ng/dL						
Baseline	464 (407–523)	473 (403–555)		33 (24–46)	32 (27–38)	
26 Weeks	464 (419–512)	450 (379–539)		32 (24–43)	32 (26–38)	
§Between group differences in ∆	0.97 (0.83–1.15)		0.79	1.33 (1.10–1.61)		0.79
Free Testosterone, pg/mL						
Baseline	46.5 (41.6–51.9)	52.2 (42.1–64.1)		7.8 (6.7–9.1)	8.6 (7.8–9.4)	
26 Weeks	46.9 (41.2–53.5)	43.8 (38.0–50.4)		8.3 (7.0–9.9)	8.7 (7.8–9.5)	
§Between group differences in ∆	0.89 (0.76–1.04)		0.13	0.98 (0.82–1.17)		0.84
SHBG, *μ*g/dL						
Baseline	2.61 (2.07–3.28)	2.85 (2.11–3.85)		4.71 (3.52–6.23)	3.70 (2.80–4.85)	
26 Weeks	2.63 (1.95–3.52)	2.55 (1.89–3.49)		4.61 (3.42–6.11)	3.78 (2.88–5.00)	
§Between group differences in ∆	0.89 (0.77–1.04)		0.16	1.03 (0.91–1.19)		0.55
Estradiol, pg/mL						
				12.6 (9.6–16.9)	11.1 (9.8–12.8)	
				11.8 (9.2–15.0)	10.8 (9.7–11.9)	
^ ^				1.02 (0.85–1.21)		0.79
IGF‐1, *μ*g/L						
Baseline	133 (107–165)	152 (125–186)		92 (74–112)	97 (74–128)	
26 Weeks	127 (111–148)	262 (210–330)		86 (75–98)	184 (158–219)	
§Between group differences in ∆	1.91 (1.50–2.41)		0.001	2.0 (1.68–2.38)		0.001

Baseline and week 26 data are unadjusted values expressed as arithmetic mean (age) or geometric means (95% CI) (LH, Total T, Free T, SHBG, Estradiol, and IGF‐1); Δ, difference in post and pretreatment values are adjusted for age, baseline value, and treatment group. Differences in mean Δ between the treatment groups is expressed as the ratio ^§^(95% CI) of the adjusted geometric means of Δ. *n*, number of subjects; *P* values indicate significance for comparisons between the GH and placebo‐treated groups.

### Effects of GH on sex steroids and LH pulsatility

**Figure 1:**
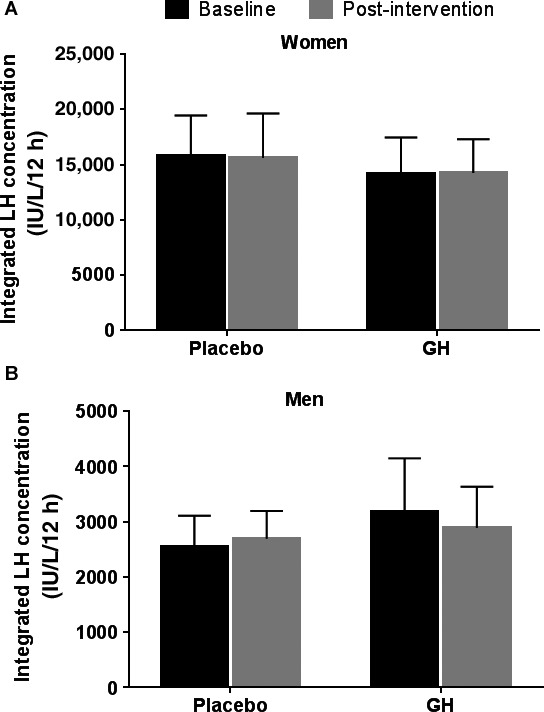
Effects of GH administration on nocturnal integrated LH concentration. Integrated serum LH concentration from q 20 min sampling (8 pm–8 am) at baseline and after 26 week of hormone administration in healthy older men (A) and women (B). Values are mean (95% CI).

As expected, GH administration significantly increased serum IGF‐1 levels in women and men. GH administration exerted no significant effects on levels of sex steroids (TT, fT, or E2), AM LH, or SHBG in older women or men (Table 1). LH secretory dynamics, including burst frequency, mass per burst, LH pulse amplitude, pulsatile and total LH production, and approximate entropy (orderliness) were not altered by treatment with GH (Table 2). No changes in integrated LH secretion in women or men were seen after 26 weeks of GH (Fig. 1).

**Table 2:** LH deconvolution parameters at baseline and after 26 weeks of GH administration in healthy older men and women

	Men			Women		
	Placebo (*n* = 12)	GH (*n* = 12)	*P* value	Placebo (*n* = 12)	GH (*n* = 12)	*P* value
Basal Secretion, IU.L^−1^.12 h^−1^						
Baseline	20.1 (16.6–24.5)	25.3 (16.6–37.7)		156 (121–198)	144 (113–186)	
26 Weeks	17.9 (11.9–27.1)	22.9 (14.7–35.1)		149 (109–204)	135 (107–169)	
Between group differences in ∆	1.00 (0.64–1.55)		0.98	0.96 (0.78–1.17)		0.68
Burst frequency, number/12 h						
Baseline	5.4 (4.6–6.4)	5.4 (4.8–5.9)		7.1 (5.6–9.0)	6.9 (5.6–9.0)	
26 Weeks	5.9 (4.9–7.2)	5.9 (5.0–6.9)		7.5 (6.9–8.2)	7.9 (7.2–8.8)	
Between group differences in ∆	1.05 (0.84–1.30)		0.64	1.07 (0.94–1.22)		0.27
Mass/burst, IU.L^−1^						
Baseline	3.0 (2.5–3.7)	3.3 (2.6–4.3)		9.5 (6.9–13.0)	8.6 (6.2–11.8)	
26 Weeks	3.1 (2.3–4.0)	2.9 (2.3–3.9)		8.8 (6.7–12)	9.1 (7.1–11.6)	
Between group differences in ∆	0.86 (0.66–1.13)		0.28	1.10 (0.90–1.34)		0.31
Amplitude, IU.L^−1^.min^−1^						
Baseline	0.20 (0.17–0.25)	0.23 (0.18–0.29)		0.65 (0.47–0.90)	0.60 (0.43–0.82)	
26 Weeks	0.21 (0.16–0.28)	0.25 (0.15–0.42)		0.61 (0.46–0.81)	0.63 (0.49–0.81)	
Between group differences in ∆	0.98 (0.61–1.56)		0.95	1.10 (0.89–1.36)		0.31
Pulsatile production rate, IU.L^−1^.12 h^−1^						
Baseline	16.2 (11.9–22.1)	17.8 (14.2–22.4)		68.0 (44.7–102.5)	59.7 (41.6–84.7)	
26 Weeks	18.5 (13.4–25.5)	17.6 (13.7–22.6)		67.2 (51.4–87.3)	72.9 (54.0–97.5)	
Between group differences in ∆	0.91 (0.65–1.28)		0.58	1.23 (0.93–1.64)		0.13
Total production rate, IU.L^−1^.12 h^−1^						
Baseline	36.9 (28.9–46.5)	43.8 (31.8–60.3)		230 (179–295)	210 (170–259)	
26 Weeks	39.6 (32.8–48.4)	42.0 (32.5–55.1)		223 (170–290)	211 (171–262)	
Between group differences in ∆	0.92 (0.80–1.06)		0.25	1.07 (0.97–1.18)		0.16
Mean, IU.L^−1^						
Baseline	3.3 (2.6–4.1)	4.0 (2.9–5.5)		20.5 (16.1–26.0)	18.5 (15.0–22.9)	
26 Weeks	3.6 (2.9–4.3)	3.8 (2.9–4.9)		19.9 (15.3–26.0)	18.7 (15.2–23.1)	
Between group differences in ∆	0.90 (0.80–1.02)		0.10	1.06 (0.97–1.17)		0.19
Approximate entropy (ApEn)						
Baseline	0.91 (0.81–1.01)	0.88 (0.76–1.00)		1.00 (0.80–1.25)	1.04 (0.92–1.18)	
26 Weeks	0.93 (0.84–1.03)	0.86 (0.76–0.96)		1.01 (0.89–1.16)	1.04 (0.92–1.20)	
Between group differences in ∆	0.92 (0.79–1.07)		0.27	1.02 (0.85–1.22)		0.24

Baseline and week 26 data are unadjusted values expressed as geometric means (95% CI); Δ, difference in post and pretreatment values are adjusted for age, baseline value, and treatment group. Differences in mean Δ between the treatment groups is expressed as the ratio (95% CI) of the adjusted geometric means of Δ. *n*, number of subjects; *P* values indicate significance for comparisons between the GH and placebo‐treated groups.

## Discussion

In this study, we evaluated the effects of 26 weeks of exogenous GH administration on nocturnal LH pulsatile dynamics and morning levels of SSs in a well‐characterized group of healthy, aged men and women with low‐normal IGF‐1 levels. We found that GH exerted no significant effects on levels of SS, SHBG, or LH, or on LH secretory dynamics, in either older women or older men. Unlike in the young (Genazzani et al. 1993), we did not find a significant stimulatory effect of exogenous GH treatment on pulsatile LH secretion or on gonadal steroid hormone production in the elderly.

All participants in this study had low normal IGF‐1 levels prior to GH administration. Furthermore, reproductive hormone concentrations in our cohort were similar to previously reported averages in healthy, elderly populations (Gray et al. 1991; Hiney et al. 1996; Hall et al. 2000; Muller et al. 2003; Divall et al. 2010). Age‐related decline in GH and IGF‐1 levels may contribute to elevated SHBG levels in men (Veldhuis et al. 1992). However, we did not see any effect of GH administration on SHBG levels in men or women in this study. In young women, IGF‐1 not only acts on theca cells of the ovary to promote androgen production, but also acts synergistically with FSH to increase aromatase activity, thereby stimulating estrogen production (Park et al. 2002). Based on our results in older women, however, IGF‐1 does not have such stimulatory effects on the postmenopausal ovary. Likewise, in men, steroidogenic (Leydig) cells of the testes express IGF‐1 receptors, and IGF‐1 acts synergistically with LH to promote testicular androgen production (Laron 1999). In our cohort of older men, however, exogenous GH had no effect on testosterone levels despite normalization of IGF‐1 levels. This suggests that the ability of the gonads to respond to stimulatory signals, particularly those downstream from GH, diminishes with age.

Levels of SS [estradiol (women) or testosterone (men)] decline progressively with age, leading to increased release of pituitary gonadotropins (Veldhuis et al. 1992; Burger 1999) secondary to the lack of negative feedback. However, reactivity of the hypothalamic‐pituitary response to declining SS seems to be muted in elderly populations, such that the LH response is attenuated compared to that which is seen in younger individuals with similarly low gonadal steroid levels (Veldhuis et al. 1994, 2001; Park et al. 2002). Levels of SSs and LH pulsatility were unaltered by GH administration in this study. This suggests that GH‐mediated increases in IGF‐1 does not affect the pituitary or the hypothalamus to modulate LH pulsatility or LH secretion.

There are many limitations of our study. LH secretory dynamics was measured at baseline and after 26 weeks of GH administration. Thus, any early effects of GH on the HPG axis may have been missed. Second, the study objective of this study was a secondary objective in the original study. Thus, the findings need to be confirmed by a larger study powered to detect significant changes. Nonetheless, there are several strengths of this study. Few studies have examined the effects of GH on LH pulsatility (Divall et al. 2010). First, this is the first study to examine the effects of GH on LH pulsatility in older individuals including men and women. Second, the effects were evaluated using a robust study design; a placebo‐controlled, double‐dummy design and studied with frequent blood sampling overnight before and during the 6th month of GH treatment. Third, in contrast to the small sample size (*n* = 19 women) of the prior study (Divall et al. 2010), a sample size (*n* = 48) is sufficiently large to detect changes in LH secretion and the lack of GH effect is unlikely due to lack of power. Finally, in the prior study the duration of GH administration was rather short (7 days), in contrast to our study where GH was administered for 6 months.

In summary, in this randomized, placebo‐controlled trial in well‐characterized, healthy older men and women, we found that at the end of 6 months of treatment with GH, at doses sufficient to normalize serum IGF‐1 levels, did not alter pulsatile secretory patterns of LH, or morning levels of SS (testosterone or estradiol) or SHBG. To our knowledge, there are no previous studies examining the effects of GH administration on the HPG axis in the elderly. In younger individuals, it is well‐established that GH administration is stimulatory to the reproductive axis, and it has been used to achieve puberty or enhance fertility (Wilson 2001) (Chandrashekar et al. 2004; Giampietro et al. 2009). On the contrary, we found here that in older individuals, the HPG axis does not respond to stimulatory cues from exogenous GH despite normalization of low‐normal baseline IGF‐1 levels.

## Conflict of Interest

The authors have nothing to disclose.
